# Haploid transcriptome analysis reveals allelelic gene expression variants, co-expressed gene groups, and linkages between expression and copy number variation

**DOI:** 10.1186/1753-6561-5-S7-O47

**Published:** 2011-09-13

**Authors:** Jukka-Pekka Verta, Sebastien Caron, Isabelle Giguère, Brian Boyle, Christian R Landry, John MacKay

**Affiliations:** 1 Centre d’étude de la forêt, Université Laval, Québec QC, Canada; 2 Department of Biology, Université Laval, Québec QC, Canada

## Background

Genetic variation can cause changes in gene expression (mRNA abundance) among individuals. This so-called heritable variation in gene expression is affected by genetic variants that are co-segregating with the gene locus (local/*cis* effects) and/or segregating independently from it (distant/*trans* effects). Genetic variation in gene expression can be measured to estimate the extant of variation in gene expression within a population, and to determine to what degree expression alleles of different genes are connected within regulatory networks. Furthermore, determining whether variation in the expression of a gene is linked to local or distant effects allows us to make inferences about how heritable variation may change depending on gene function, the number of interacting partners, genetic architecture and evolutionary history [1,2].

Detecting heritable variation in gene expression can be a challenging task in diploid organisms, mainly because of tissue-specificity and dominance effects of allelic expression. For example, up to 70% of gene expression alleles in *Drosophila* may be masked by dominance [3]. We developed an experimental system to overcome these obstacles by utilizing the conifer seed’s maternally derived haploid tissue, the megagametophyte. Analyzing a set of sibling megagametophytes allows us to first, measure separately the expression each of the two alleles in the maternal genome in the absence of dominance and second, identify genes whose expression levels are co-segregating. In addition, the megagametophyte allows us to categorize the underlying genetic variants into local or distant with a simple co-segregation assay.

## Methods

We set out to characterize segregating variation in gene expression in white spruce (*Picea glauca* [Moench] Voss). We analyzed the transcriptomes of germinating sibling megagametophytes from two controlled-crossed families (C9412516: male 2388 x female 77111 & C9612856: male 80109 x female 80112) with a custom microarray comprised of 32,000 spotted oligonucleotides, which represent over 25,000 unique white spruce genes. Each megagametophyte was split into two halves to provide technical replicates that were analyzed separately. A separate comparison of microarray results and RNA-Seq data has been carried out to validate the quality of the microarray.

The single-color microarray data was background-corrected and quantile-normalized with the R package Limma [4]. We used the R package Mclust [5] to test for unimodal vs. bimodal expression distributions of each gene across sibling megagametophytes. Genes that exhibited at least two expression alleles, which segregated within the 95% IC of their expected proportions, were selected. The segregation had to be repeatable with a replicate sample set to be considered valid.

## Results and discussion

Analysis of two families of sibling megagametophytes (n=18) identified close to a thousand genes with segregating gene expression patterns in both of the two families. Approximately 10% of these genes were shared. Zero replicates are expected to have the same clustering pattern by chance alone (binomial *p*≈*3.810-6*| n=18). The number of variable genes is comparable to that found between 50 nearly isogenic *Drosophila* lines [6].

We have discovered a large number of genes with gene expression patterns segregating in a Mendelian way in white spruce. We are presently analyzing the relationships between gene function and paralog number with the variation in its expression in order to determine whether heritable variation in gene expression is associated with same genetic attributes in white spruce as what has been reported in model organisms. We have also begun investigating the contribution of local vs. distant effects on the expression alleles identified in the megagametophytes, and their nature, by studying the co-segregation of gene expression, SNPs and copy number variations (CNVs). Preliminary comparative genomic hybridization data suggests a significant portion of genes which show segregating gene expression alleles also exhibit CNVs. In follow-up experiments, we will address the amount of dominance between the gene expression alleles by comparing gene expression in megagametophytes versus self-fertilized embryos.
